# Optimizing pregnancy management of chronic hypertension: A case report of cerebrovascular accident in a pregnant woman and literature review

**DOI:** 10.1097/MD.0000000000042649

**Published:** 2025-06-06

**Authors:** Chunfei Wang, Qiang Wei

**Affiliations:** a Department of Obstetrics and Gynecology, Key Laboratory of Birth Defects and Related Diseases of Women and Children, Ministry of Education, West China Second University Hospital, Sichuan University, Chengdu, People’s Republic of China.

**Keywords:** antihypertensive drugs, cerebrovascular accident, chronic hypertension, pregnancy management, severe preeclampsia

## Abstract

**Rationale:**

The prevalence of chronic hypertension among pregnant women is steadily increasing worldwide. Besides chronic hypertension is a major risk factor for preeclampsia (PE). Therefore, the standardization of monitoring and management – focusing on early identification and the judicious use of antihypertensive medications to improve maternal and fetal outcomes – has become a critical concern for clinicians.

**Patient concerns:**

A 31-year-old pregnant woman with chronic hypertension exhibited repeated blood pressure of 160/110 mm Hg or higher throughout her pregnancy, and there were no laboratory or clinical signs indicative of PE. Fluctuations in her blood pressure resulted in an acute cerebrovascular accident during the period of expectant management.

**Diagnoses:**

Chronic hypertension with severe PE.

**Interventions:**

The patient was admitted due to acute severe hypertension and received corticosteroids for fetal lung maturation. Expectant management was initially considered due to relatively stable condition during the first 3 days of hospitalization. However, on the 4th day, despite treatment with both oral and intravenous antihypertensive agents, blood pressure remained uncontrolled, even reaching 200/105 mm Hg or higher. Unfortunately, the persistent severe hypertension resulted in an acute stroke, necessitating emergency cesarean section and neurosurgical intervention at 31 + 5 weeks of gestation.

**Outcomes:**

The neonate was transferred to the Neonatal Intensive Care Unit and remained there for 38 days before discharge. The patient was discharged by postoperative day 19 and continued with rehabilitation treatment. At the latest follow-up, muscle strength in her left limb remained at grade 1, with no significant recovery observed.

**Lessons:**

Pregnant women with chronic hypertension who present persistently elevated and uncontrollable blood pressure should be diagnosed with severe PE. Treatment regimens for hypertensive disorders of pregnancy typically involve oral antihypertensive medications, which should be administered judiciously based on clinical guidelines and trial evidence. For chronic maintenance treatment, labetalol and extended-release nifedipine are recommended as 1st-line agents by most international guidelines, with nifedipine being a viable initial option for severe hypertension. For women with severe PE, after corticosteroids have been administered for fetal lung maturation, an overall assessment of maternal and placental-fetal conditions should guide whether expectant management is feasible or if prompt delivery is required to minimize severe complications.

## 1. Introduction

The estimated prevalence of chronic hypertension in pregnant women is currently about 3% to 5%, which continues to rise every year due to factors such as advancing maternal age, lifestyle changes, and increasing rates of obesity.^[1]^ A 2011 epidemiological survey in China reported a 5.22% incidence of hypertensive disorders of pregnancy (HDP), with chronic hypertension accounting for 6% of cases, and chronic hypertension with superimposed preeclampsia (PE) for 3.68%.^[2]^ Although recent large-scale data on pregnant women with chronic hypertension are limited, available evidence points to a growing trend. A recent study from the United States found that the incidence of chronic hypertension in pregnancy in 2018 was at least double that of a decade prior, indicating a consistent upward trend.^[3]^

Chronic hypertension is widely recognized as a major risk factor for PE. Between 20% and 50% of women with chronic hypertension may develop superimposed PE, which tends to present earlier and with more severe consequences than in women without hypertension.^[4]^ Given the increasing prevalence of chronic hypertension in pregnancy, the need for effective surveillance and management has become critical to improving maternal and perinatal outcomes. Of particular importance are the early detection of changes in a patient’s condition and the standardized use of antihypertensive medications to mitigate those risks.

In clinical practice, delayed diagnosis and intervention for complications related to chronic hypertension, as well as inconsistent use of antihypertensive medications, have contributed to adverse maternal and fetal outcomes. Here, we reported a case of a pregnant woman with chronic hypertension complicated by severe PE who experienced poor maternal and fetal outcomes. This case is accompanied by a literature review aimed at optimizing the management of chronic hypertension during pregnancy. This study was approved by the ethics committees at the West China Second University Hospital of Sichuan University. Written informed consent was obtained from the patient and her partner before the procedure and manuscript publication.

## 2. Case presentation

A 31-year-old nulliparous Chinese woman (G3P0 + 2) at 17 weeks of gestation was admitted to our hospital with a sudden onset of elevated blood pressure (166–191/99–115 mm Hg). During previous regular outpatient checkups, she had no laboratory abnormalities results or clinical symptoms indicative of HDP. Upon admission, she was immediately treated with magnesium sulfate for spasm and antihypertensive medications, including labetalol (100 mg every 8 hours), extended-release nifedipine (30 mg every 12 hours), and intravenous urapidil to control her blood pressure. Following acute treatment, her blood pressure stabilized (116–153/68–100 mm Hg), and repeat testing of urine, blood, coagulation function, as well as liver and kidney function yielded normal results. The patient was then discharged and advised to continue regular outpatient follow-ups with long-term oral antihypertensive medication.

During routine outpatient checkups throughout the pregnancy, the patient’s blood pressure was continuously monitored, and her antihypertensive regimen was adjusted to oral labetalol (200 mg every 8 hours) and extended-release nifedipine (30 mg every 12 hours) based on blood pressure readings, which mostly remained within the normal range. Routine urinalysis showed protein levels ranging from negative to 2+, with the protein/creatinine ratio of 0.14 to 0.22, and a 24-hour urine protein level of 184 mg. Additional tests, including the fetal nuchal translucency test, 1st and 2nd-trimester screening tests for Down syndrome, a 75-g oral glucose tolerance test, thyroid function tests, fetal cardiac ultrasound, and fetal systemic ultrasound were all performed and were normal. However, an ultrasound at 28 weeks showed the fetal femur length was below the 10th percentile for gestational age. After prenatal genetic counseling and diagnosis evaluation, dynamic monitoring of femur length was initiated, and amniocentesis was subsequently performed.

Within 4 hours after the amniocentesis, the patient’s blood pressure remained persistently elevated at 160/100 mm Hg or higher, although she did not experience dizziness, headache, or visual disturbances. Magnesium sulfate, along with 30 mg of extended-release nifedipine and intravenous nitroglycerin, was administered immediately to lower her blood pressure. Upon readmission to the hospital with the diagnosis of chronic hypertension in pregnancy, and G3P0 + 2 intrauterine pregnancy at 29 + 2 weeks, the patient’s vital signs were stable, and obstetric examination revealed an abdominal circumference of 93 cm, uterine height of 26 cm, fetal heart rate of 140 beats/min, and no palpable contractions. After readmission, corticosteroids were promptly administered to promote fetal lung maturation, while blood pressure was managed with labetalol, extended-release nifedipine, methyldopa, and additional intravenous urapidil and nitroglycerin as needed. The treatment process is detailed in Table 1.

**Table 1:** Detailed treatment process.

Weeks of gestation	Antihypertensive agents					Magnesium sulfate†	Blood pressure(mm Hg)	Treatment notes
	Labetalol (dose/frequency)	Extended-release nifedipine (dose/frequency)	Methyldopa (dose/Q 12 h)	Intravenous urapidil*	Intravenous nitroglycerin*			
29 + 2	200 mg Q 8 h	30 mg Q 12 h			ⅹ	√	138–165/81–92	Corticosteroids were administered
29 + 3	200 mg Q 8 h	30 mg Q 12 h				√	137–165/85–105	Corticosteroids were administered
29 + 4	200 mg Q 6 h	30 mg Q 12 h				√	138–161/78–94	
29 + 5	200 mg Q 6 h	30 mg Q 12 h		√		ⅹ	142–157/74–98	10 mg oral nifedipine at 17:00 when blood pressure was 158/99 mm Hg; magnesium sulfate and intravenous urapidil were administered at 21:16 when blood pressure was 180/100 mm Hg
29 + 6	200 mg Q 6 h	30 mg Q 12 h		√	√	√	145–162/81–94	Intravenous nitroglycerin was administered when blood pressure fluctuated between 160–180 and 80–100 mm Hg since 21:34
30	200 mg Q 6 h	30 mg Q 12 h	250 mg	√	ⅹ	√	129–165/81–100	After 03:03, blood pressure ranged between 127–142 and 78–89 mm Hg, leading to the discontinuation of intravenous nitroglycerin
30 + 1	200 mg Q 6 h	60 mg/d	250 mg	√		√	140–150/90–95	
30 + 2	200 mg Q 6 h	60 mg/d	250 mg	√		√	134–159/68–97	
30 + 3	200 mg Q 6 h	60 mg/d	500 mg	√		√	131–163/77–101	
30 + 4	200 mg Q 6 h	60 mg/d	500 mg	ⅹ		√	133–158/75–96	Blood pressure stabilized, and intravenous urapidil was terminated
30 + 5	200 mg Q 6 h	60 mg/d	500 mg			√	131–159/81–92	
30 + 6	200 mg Q 6 h	60 mg/d	500 mg	√	√	ⅹ	139–187/80–114	During the night, the patient exhibited significant emotional distress and severe anxiety, with blood pressure fluctuating between 179–203 and 104–110 mm Hg. Intravenous nitroglycerin was added alongside urapidil to lower blood pressure further
31	200 mg Q 6 h	60 mg/d	500 mg	√	ⅹ		132–160/76–100	
31 + 1	200 mg Q 6 h	60 mg/d	500 mg	√			126–157/73–91	After a cardiology consultation, it was advised to rule out secondary hypertension due to the challenges in managing blood pressure with multiple medications. However, all relevant investigations revealed no significant abnormalities
31 + 2	200 mg Q 6 h	60 mg/d	500 mg	√	√		125–163/78–94	Blood pressure readings fluctuated between 189–200 and 107–122 mm Hg throughout the night, starting at 22:10. Intravenous nitroglycerin was added alongside urapidil to further reduce blood pressure
31 + 3	200 mg Q 6 h	60 mg/d	500 mg	√	ⅹ		133–160/75–104	
31 + 4	200 mg Q 6 h	60 mg/d	500 mg	√			125–176/77–114	
31 + 5	200 mg Q 6 h	60 mg/d	500 mg	√	√			At 14:25, the blood pressure was 174/113 mm Hg, an increase in urapidil dosage and 60 mg oral extended-release nifedipine was administered immediately. By 15:42, blood pressure had risen to 202/122 mm Hg, and intravenous nitroglycerin was added to lower blood pressure. However, at 15:51, blood pressure escalated to 206/123 mm Hg, accompanied by the sudden onset of delirium, agitation, and progressive loss of consciousness. An emergency cesarean section was conducted at 17:08

* Urapidil infusion rate (3–40 mg/h) and nitroglycerin infusion rate (1–7 mg/h) were titrated according to real-time blood pressure monitoring, followed by protocol-guided tapering to discontinuation upon achieving and maintaining normotensive thresholds.

† Magnesium sulfate infusion rate (0.74–1.48 g/h) was titrated based on serum magnesium concentrations.

In the subsequent days, blood pressure remained unstable, though multiple urinalyses indicated negative proteinuria and 24-hour urine protein levels decreased from 294 to 50 mg. Routine blood analysis, along with tests of coagulation, liver and kidney function were normal. Echocardiography and color Doppler imaging of the liver, bile ducts, pancreas, spleen, and urinary tract showed no significant abnormalities. However, on day 18 of hospitalization, at 31 + 5 weeks of gestation, the patient’s blood pressure rapidly escalated from 175/113 to 206/123 mm Hg within approximately 1.5 hours, accompanied by the sudden onset of delirium, agitation, and progressive loss of consciousness. The multidisciplinary team suspected intracranial hemorrhage and diagnosed chronic hypertension with severe PE. An emergency cesarean section was conducted following a 4 g loading dose of intravenous magnesium sulfate. The surgery successfully delivered a live infant weighing 1600 g, with Apgar scores of 6, 8, and 8 at 1, 5, and 10 minutes, respectively. Due to preterm birth, the neonate was transferred to the Neonatal Intensive Care Unit and remained there for 38 days before discharge, weighing 2480 g. Besides, approximately 600 mL of clear amniotic fluid was noted during the surgery.

After surgery, an immediate postoperative cranial CT showed a hyperdense mass in the right insular, basal ganglia, and external capsule region with perihematomal edema, indicative of cerebral parenchymal hemorrhage. Hyperdense areas were also identified in both lateral ventricles, suggesting intraventricular hemorrhage. The neurosurgery team further conducted a 3-dimensional enhanced CT of the head and neck and confirmed a right basal ganglia hemorrhage with a hematoma measuring approximately 3.0 × 5.7 cm. The hematoma had extended into the ventricular system, resulting in intraventricular bleeding and compression of the right lateral ventricle. Emergency surgical intervention was performed and went well, including craniotomy hematoma removal from the right basal ganglia, cerebrospinal fluid leak repair, and decompressive craniectomy.

Postoperatively, the patient received supportive care, including oral metoprolol (47.5 mg daily) and valsartan–amlodipine tablets (I; 80 mg valsartan and 5 mg amlodipine daily) for blood pressure management. Eleven days after the surgery, her consciousness had notably improved, as she responded to verbal stimuli, opened her eyes spontaneously, and followed motor commands. Motor function in her right limbs was normal, while muscle strength in the left limbs was assessed as grade 1 on the British Medical Research Council scale. The patient was discharged 19 days after the surgery and continued with rehabilitation treatment. At the latest follow-up, muscle strength in her left limb remained at grade 1, with no significant recovery observed.

## 3. Discussion

### 3.1. Background

Hypertensive disorders during pregnancy are one of the leading causes of maternal and perinatal mortality worldwide. Numerous studies have shown that chronic hypertension in pregnancy is closely associated with adverse maternal and perinatal outcomes. Pregnant women with chronic hypertension have a 5 to 6 times higher risk of developing peripartum cardiomyopathy, cerebrovascular accidents, kidney failure, pulmonary edema, and even death compared to normotensive pregnant women. Additionally, pregnant women with chronic hypertension are more likely to experience preterm delivery, fetal growth restriction, and neonatal mortality compared to healthy pregnant women.^[4–7]^ Moreover, chronic hypertension is a high-risk factor for PE, which tends to occur earlier and with more severe complications, such as cerebrovascular accident, spontaneous hepatic rupture and so on. The crucial points are the atypical clinical presentation of PE, given that these severe complications can occur without clinical signs or prodromal symptoms.^[8,9]^ Given that this cohort of women with chronic hypertension will inevitably encounter pregnancy and childbirth, optimal management during the prenatal and perinatal periods is essential.

### 3.2. Definition and diagnosis of chronic hypertension

Chronic hypertension in pregnancy is defined as elevated blood pressure (systolic pressure ≥ 140 mm Hg and/or diastolic pressure ≥ 90 mm Hg) present either before conception or before 20 weeks of gestation, confirmed by at least 2 measurements taken at least 4 hours apart. This condition persists beyond 12 weeks postpartum, distinguishing it from gestational hypertension, which develops after 20 weeks and resolves after delivery.^[10]^ In this case, the patient had no prior history of hypertension, but her blood pressure consistently exceeded 140/90 mm Hg at 17 weeks of gestation and remained elevated for over 12 weeks postpartum, confirming the diagnosis of chronic hypertension in pregnancy.

Chronic hypertension with PE is generally regarded as an exclusive diagnosis. Differentiating superimposed PE from chronic hypertension in pregnant patients with elevated blood pressure in pregnancy is often challenging – especially with a sudden increase in baseline blood pressure. Careful surveillance of symptoms associated with PE is essential, and diagnosis should be based on laboratory findings. New onset of thrombocytopenia, newly developed or worsening proteinuria, elevated serum creatinine, liver function abnormalities, or other signs of organ damage associated with PE indicates the diagnosis of chronic hypertension with PE.^[10]^

The patient in this case presented with multiple urinalysis results ranging from negative to 2 + proteinuria, 24-hour urine protein levels below 300 mg and protein/creatinine ratios under 0.3 throughout her pregnancy. Despite repeated blood pressure readings of 160/110 mm Hg or higher, there were no laboratory or clinical signs indicative of PE. Therefore, at 29 + 2 weeks of gestation, during her 2nd admission, she was diagnosed with chronic hypertension with elevated blood pressure. However, despite treatment with 3 to 4 antihypertensive medications, the patient’s blood pressure remained poorly controlled. From a practical point of view, PE should be considered 1st. International guidelines clearly state that persistent and uncontrolled hypertension is indicative of chronic hypertension with severe PE.^[10,11]^ However, this critical point can sometimes be overlooked by clinicians, potentially resulting in delays in diagnosis and subsequent treatment.

### 3.3. Initial threshold and target blood pressure for antihypertensive treatment

Although antihypertensive treatment is universally recommended for severe chronic hypertension (systolic pressure ≥ 160 mm Hg or diastolic pressure ≥ 110 mm Hg) and PE, controversy exists regarding the threshold for initiating treatment during pregnancy. At the 2024 Annual Clinical and Scientific Meeting of the American College of Obstetricians and Gynecologists (ACOG), it was recommended that antihypertensive treatment be initiated at 140/90 mmHg for pregnant women with chronic hypertension. This recommendation is based on a recent randomized controlled trial by Tita et al,^[12]^ which demonstrated that starting treatment at a threshold of 140/90 mm Hg, as opposed to waiting for severe hypertension, was associated with improved maternal and perinatal outcomes. Furthermore, patients who are stabilized on antihypertensive treatment in early pregnancy should continue their medication to maintain adequate blood pressure control, as poor control can lead to severe hypertension and adverse maternal and fetal outcomes.

The optimal target blood pressure level during pregnancy for women receiving therapy for chronic hypertension remains debated.^[10]^ A 2015 study published in the *New England Journal of Medicine*, which included 987 participants with HDP (74.6% of whom had chronic hypertension), found a reduced risk of severe hypertension and improved perinatal outcomes associated with maintaining diastolic blood pressure strictly below 85 mm Hg, compared to the traditional target of that ≤100 mm Hg.^[13]^ Consequently, the International Society for the Study of Hypertension in Pregnancy recommends maintaining systolic blood pressure 110–140 mm Hg and diastolic pressure 80–85 mm Hg for pregnant women with chronic hypertension on antihypertensive therapy.^[14]^ Furthermore, the National Institute for Health and Care Excellence suggests targeting blood pressure levels below 135/85 mm Hg.^[15]^ However, excessively lowering blood pressure can be harmful, as it may lead to hypoperfusion of critical areas such as the maternal brain, heart, or placenta. Therefore, blood pressure management should aim for stability, with minimal fluctuations. A target reduction of 10% to 25% of the mean arterial pressure is deemed optimal, with stabilization typically achieved within 24 to 48 hours.^[16]^ In this case, the pregnant woman experienced adverse events, including a cerebrovascular accident and cerebral hemorrhage, probably due to significant fluctuations in blood pressure during antihypertensive treatment.

### 3.4. Standardized selection and application of antihypertensive agents

Oral antihypertensive medication is the cornerstone of long-term treatment for pregnant women with HDP. Most international guidelines recommend labetalol and nifedipine as 1st-line agents.^[10,11]^ According to ACOG, the most commonly used oral antihypertensive medications in pregnancy include labetalol, nifedipine, methyldopa, and hydrochlorothiazide. Labetalol is typically initiated at an oral dose of 200 to 400 mg/d, with a maximum dose of 2400 mg. Extended-release nifedipine is initiated at an oral dose of 30 to 60 mg/d, with a maximum dose of 120 mg. Methyldopa is initiated at an oral dose of 500 to 750 mg/d, with a maximum dose of 3000 mg. These agents can be used individually or in combination for chronic blood pressure management. In addition, based on the 2021 Chinese expert consensus on blood pressure management during pregnancy, if blood pressure remains poorly controlled with monotherapy or combination therapy of labetalol and extended-release nifedipine, methyldopa can be added at a dose of 250 to 500 mg, taken 2 to 3 times/d.^[17]^

Methyldopa has been the most widely used alpha-adrenergic agonist for managing HDP since the 1960s. Notably, it is the only antihypertensive medication during pregnancy designated as category B by the Food and Drug Administration, whereas other commonly used agents, such as labetalol, β-blockers, calcium channel blockers, and thiazide diuretics, are designated as category C.^[1,18]^ However, the use of methyldopa has declined in recent years, with a growing preference for labetalol and nifedipine.^[19]^ This shift aligns with findings from recent studies, which indicate that methyldopa is less effective for both long-term oral antihypertensive treatment and the control of severe hypertension. Moreover, it is associated with several potential side effects, including headache, dizziness, muscle pain, weakness, swelling, dry mouth, and symptoms of depression, particularly when initiating the medication or increasing the dose.^[18,20,21]^ Our patient was initially treated with labetalol (200 mg every 8 hours) and extended-release nifedipine (30 mg every 12 hours) before her 2nd hospitalization. Due to inadequate blood pressure control, her labetalol dosage was increased to 200 mg every 6 hours, and methyldopa (250 mg every 12 hours, later increased to 500 mg every 6 hours) was added according to Chinese guidelines. Despite these adjustments, the patient’s blood pressure remained unstable, necessitating the use of intravenous medications to assist in lowering her blood pressure intermittently. This observation mirrors the inferior efficacy of methyldopa compared to other antihypertensive agents, as reported in the relevant literature.

Most guidelines recommend oral nifedipine, intravenous labetalol, and hydralazine as 1st-line agents for the treatment of acute, severe hypertension during pregnancy.^[10,11,22]^ However, a systematic review of 35 randomized trials involving 3573 women found insufficient evidence to recommend particular drugs for the acute treatment of severe hypertension during pregnancy. Clinicians are advised to select antihypertensive medications based on familiarity, cost, and availability.^[22,23]^ Moreover, pregnant women with exacerbated hypertension have been found to exhibit impaired cerebral autoregulation, which increases their risk of cerebrovascular complications, such as stroke, hypertensive encephalopathy, and eclampsia.^[24]^ Until stronger evidence emerges, the prompt initiation of 1st-line treatments, as recommended by ACOG, within 30 to 60 minutes for severe hypertension is crucial to reduce the risk of maternal stroke.^[22]^

Although clinical trials have shown that intravenous medications are effective and widely used for lowering blood pressure, a recent trial published in *The Lancet* demonstrated that oral antihypertensives, such as labetalol, extended-release nifedipine, and methyldopa, effectively reduced blood pressure to the reference range in most pregnant women with severe hypertension. Among these, extended-release nifedipine as a single agent achieved the primary outcome more rapidly than labetalol or methyldopa.^[25]^ Another 2 network meta-analyses further supported these findings, showing that oral nifedipine had a higher success rate in reaching target blood pressure compared to intravenous labetalol and hydralazine for the acute management of severe hypertension in pregnancy.^[26,27]^ Moreover, oral nifedipine was found to significantly reduce cerebral perfusion pressure, lowering the risk of cerebrovascular complications when compared to intravenous labetalol.^[24]^ In contrast, hydralazine’s dose-response is often unpredictable, potentially leading to inconsistent blood pressure control. The use of intravenous hydralazine has been associated with a higher incidence of adverse maternal and perinatal outcomes compared to intravenous labetalol and oral nifedipine, including increased rates of maternal hypotension, placental abruption, oliguria, and poor Apgar scores.^[28,29]^ Therefore, oral nifedipine should be considered as a viable initial and preferred option for blood pressure management in pregnant women with severe hypertension.

Our patient received intravenous nitroglycerin and urapidil multiple times at intervals during hospitalization. However, their use as antihypertensive agents in the management of pregnant women with severe hypertension is uncommon. Nitroglycerin, a nitric oxide donor with effective tocolytic action and low toxicity, is primarily used as a uterine relaxant in various obstetric emergencies.^[30]^ While limited studies demonstrated its efficacy as an antihypertensive medication during pregnancy,^[31]^ no international guidelines recommend its use for this purpose, except for the Chinese guidelines.^[16]^ Urapidil, a postsynaptic α1-receptor antagonist that has been used in clinical practice for over 20 years, is known to cross the placenta, though there is insufficient toxicological data regarding its effects on the fetus and newborn.^[32]^ A few trials have shown urapidil to be effective and well-tolerated as an antihypertensive agent in pregnant women.^[33]^ As a result, Austrian, French, and German guidelines all recommended intravenous urapidil for managing HDP, while it has not been endorsed by guidelines of ACOG, National Institute for Health and Care Excellence, and the Society of Obstetricians and Gynaecologists of Canada.^[34]^ Therefore, the clinical effectiveness and safety of IV nitroglycerin and urapidil still require validation through large-scale, multicenter, prospective clinical trials to provide stronger evidence for their use as antihypertensive agents in pregnant women.

In this case, the pregnant woman was readmitted for acute severe hypertension, with systolic blood pressure fluctuating between 160 and 206 mm Hg, and diastolic pressure between 110 and 123 mm Hg during hospitalization. Although long-term oral antihypertensive agents were combined with intravenous urapidil or nitroglycerin at intervals for blood pressure management, her blood pressure remained uncontrolled with a single intravenous agent, prompting the use of a combination of urapidil and nitroglycerin. Unfortunately, the persistent severe hypertension resulted in an acute stroke, necessitating emergency surgical intervention. Notably, during the entire course of managing her acute severe hypertension, the use of oral nifedipine was insufficient. It was administered only once – 10 mg on the 4th day of her 2nd admission – when her blood pressure reached 158/99 mm Hg. This situation highlights the urgent need for further research to confirm both the efficacy and safety of intravenous urapidil and nitroglycerin in the acute treatment of severe hypertension during pregnancy. Additionally, oral nifedipine should be considered the preferred option for managing acute severe hypertension during pregnancy, with appropriate dosages administered continuously.

### 3.5. Optimal timing of delivery

There is limited data to guide the optimal timing of delivery for pregnant women with chronic hypertension.^[10]^ ACOG recommends delivery after 38 weeks of gestation for women with chronic hypertension who have no long-term antihypertensive medications and no additional maternal or fetal complications supporting earlier delivery. For those on maintenance antihypertensive medications, delivery is recommended after 37 weeks. Delaying delivery beyond 39 weeks is associated with an increased risk of PE.^[10]^ A recent study demonstrated that, for women with mild chronic hypertension (blood pressure ≤ 160/110 mm Hg), delivery at 39 weeks of gestation provides the best balance between maternal and neonatal outcomes.^[35]^

Given the limited reliable clinical trial data to guide decisions about the timing of delivery in women with chronic hypertension with PE, clinical guidelines recommend that delivery timing largely align with the management of PE.^[10]^ Additionally, there is insufficient data to make specific recommendations regarding the optimal timing of delivery for women with severe PE.^[36]^ Generally, about half of the women with early-onset severe PE do not require delivery within 48 hours of admission, allowing time for corticosteroid therapy to promote fetal lung maturation. Expectant management may be considered if maternal and fetal conditions remain stable.^[37]^ A 2018 Cochrane review, which included 6 small trials with a total of 748 patients, reported that expectant management for early-onset PE with severe features may improve neonatal outcomes. Further large-scale, high-quality trials are necessary to confirm or refute these findings and to provide reliable conclusions about maternal outcomes.^[38]^

Our literature review found that most prospective controlled trials report that expectant management can prolong pregnancy by approximately 7 to 15 days in women with early-onset severe PE. However, only one-third of these women remain pregnant beyond 7 days, and around 65% experience an extension of just 3 days.^[39]^ Additionally, this approach may increase the risk of placental abruption and small-for-gestational-age infants.^[39,40]^ Therefore, expectant management of PE with severe features before 34 weeks of gestation should be undertaken with strict patient selection and conducted in settings equipped with adequate maternal and neonatal care. Otherwise, prompt delivery after corticosteroid therapy is recommended if conditions remain unstable and show no signs of improvement despite active treatment.

Chronic hypertension with severe PE can result in acute complications for both the woman and fetus, including persistently elevated and uncontrollable blood pressure, eclampsia, pulmonary edema, coagulopathy, renal failure or injury, placental abruption, and abnormal fetal status. ACOG currently recommends immediate delivery rather than expectant management at any gestational age once maternal stabilization is achieved if any of those complications are present.^[10,11]^ In this case, the pregnant woman was readmitted due to acute severe hypertension and received corticosteroids for fetal lung maturation. Expectant management was initially considered due to satisfactory control of blood pressure and relatively stable condition during the first 3 days of hospitalization. However, on the 4th day, despite treatment with both oral and intravenous antihypertensive agents, her blood pressure remained consistently elevated at 200/105 mm Hg or higher. After her condition was stabilized with active intervention, continued expectant treatment rather than prompt delivery resulted in an acute cerebrovascular accident, complicated by cerebral hemorrhage. Accordingly, continued expectant management for women with severe PE after corticosteroid therapy should be based on a comprehensive clinical evaluation of both maternal and fetal conditions. It is not medically advisable to prolong pregnancy at the expense of the mother’s safety solely for fetal benefit.

## 4. Conclusion

In conclusion, the rising prevalence of chronic hypertension during pregnancy has prompted clinical practitioners to prioritize its impact on maternal and perinatal outcomes. There is a critical need to raise awareness about the early identification of pregnant women with chronic hypertension with severe PE, who often present with persistently elevated and unmanageable blood pressure. Given the considerations of fetal safety, the range of available antihypertensive medications is limited, making it essential to rationalize and standardize their use according to guidelines and clinical trial evidence. Labetalol and extended-release nifedipine are reasonable 1st-line options for chronic maintenance treatment of pregnant women, recommended above other antihypertensives. For acute severe hypertension in pregnancy, oral nifedipine may be the preferred choice. Concurrently, for pregnant women with chronic hypertension with severe PE who experience repeated blood pressure fluctuations, prompt delivery after corticosteroid administration is associated with better maternal and perinatal outcomes, provided that the clinical conditions allow.

## Acknowledgments

We feel grateful for the doctors and staff who have been involved in this work.

## Author contributions

**Conceptualization:** Chunfei Wang, Qiang Wei.

**Data curation:** Chunfei Wang, Qiang Wei.

**Writing – original draft:** Chunfei Wang, Qiang Wei.

**Writing – review & editing:** Qiang Wei.
